# Specific Light-Up System for Protein and Metabolite Targets Triggered by Initiation Complex Formation

**DOI:** 10.1038/s41598-017-15697-8

**Published:** 2017-11-09

**Authors:** Hiroto Fujita, Yuka Kataoka, Remi Nagano, Yasuyo Nakajima, Masanobu Yamada, Naoki Sugimoto, Masayasu Kuwahara

**Affiliations:** 1 Graduate School of Science and Technology, Gunma University, 1-5-1 Tenjin-cho, Kiryu, Gunma, 376-8515 Japan; 2 Department of Internal Medicine, Division of Endocrinology and Metabolism, Graduate School of Medicine, Gunma University, 3-39-15 Showa-machi, Maebashi, 371-8511 Japan; 3 Frontier Institute for Biomolecular Engineering Research (FIBER), Konan University, 7-1-20 Minatojima-minamimachi, Kobe, 650-0047 Japan; 4 Graduate School of Frontiers of Innovative Research in Science and Technology (FIRST), Konan University, 7-1-20 Minatojima-minamimachi, Kobe, 650-0047 Japan

## Abstract

Gene regulation systems are mimicked by simple quantitative detection of non-nucleic acid molecular targets such as protein and metabolite. Here, we describe a one-tube, one-step real-time quantitative detection methodology for isothermal signal amplification of those targets. Using this system, real-time quantitative detection of thrombin and streptomycin, which were used as examples for protein and metabolite targets, was successfully demonstrated with detection limits of at most 50 pM and 75 nM, respectively. Notably, the dynamic range of target concentrations could be obtained for over four orders of magnitude. Thus, our method is expected to serve as a point-of-care or on-site test for medical diagnosis and food and environmental hygiene.

## Introduction

Gene expressions are generally subject to thermodynamic and kinetic controls based on association and dissociation of protein transcription factors to specific sites on DNA^1,2^. Once the initiation complex forms, RNA polymerase executes the designated transcription. Transcriptions are precisely regulated and never start without meeting particular conditions^3^. This mechanism for gene expression has been used in biological techniques for analysing molecular interactions, such as one-, two-, and three-hybrid systems functioning in living cells^4–6^.

Simplified testing for specific molecular targets has been gaining traction^7–11^ in view of recent back-to-back biomarker discoveries. Here, we anticipated that further applications using alternative materials might enable the construction of a simple and sensitive biomarker detection method for *in vitro* use. Ideally, we envisioned that, as with the real-time immuno-polymerase chain reaction (RT-IPCR) method^12,13^, the presence of the target molecule can be converted into the production of amplified polynucleotide strands, which can quantitatively be detected as a fluorescent signal; however, the detection reaction isothermally proceeds in a one-tube, one-step manner, i.e., without the requirement of temperature fluctuations or washing steps.

**Figure 1:**
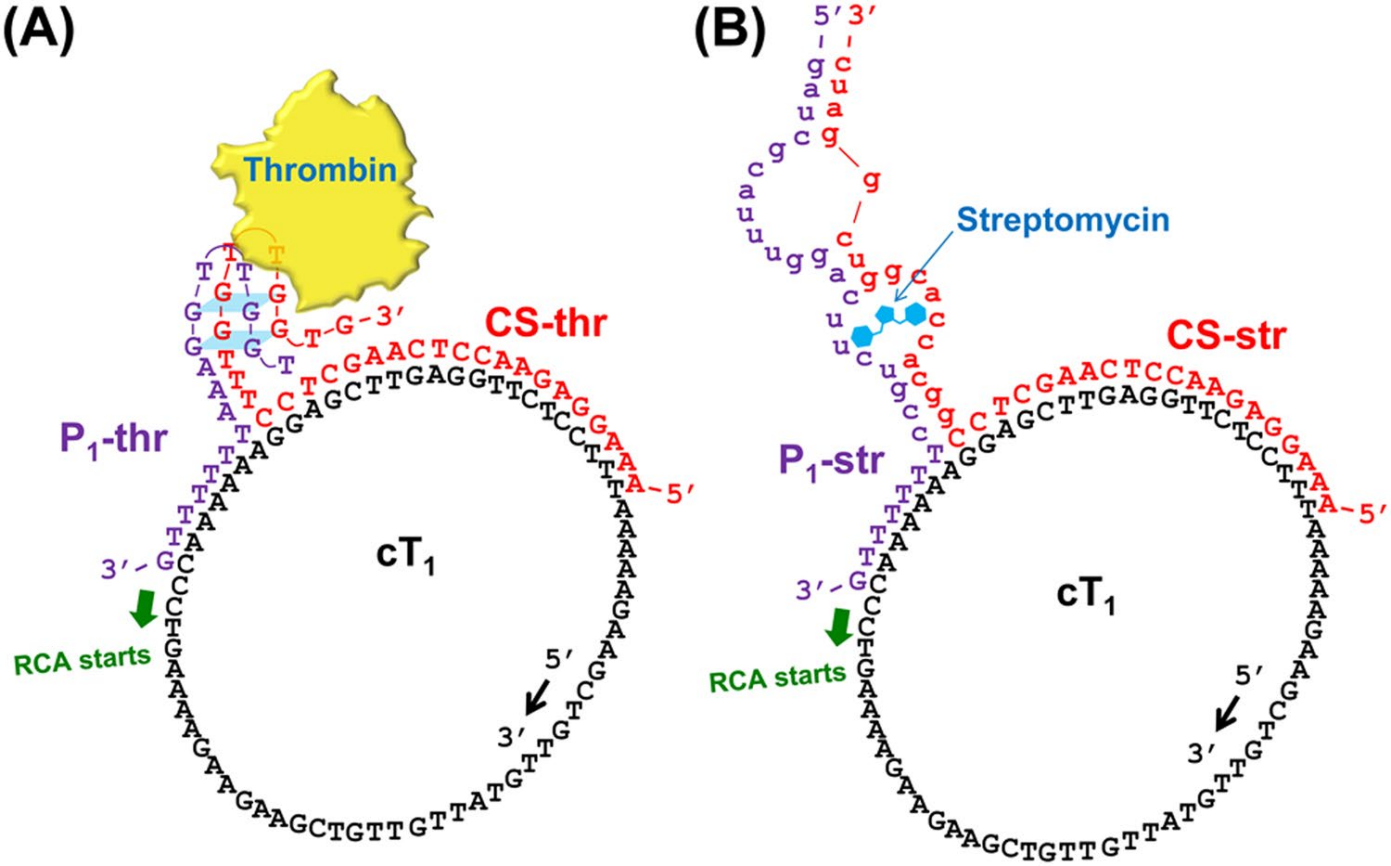
Illustration of the four-membered initiation complexes for specific detections of (**A**) human thrombin and of (**B**) streptomycin on the basis of the SATIC methodology.

**Figure 2:**
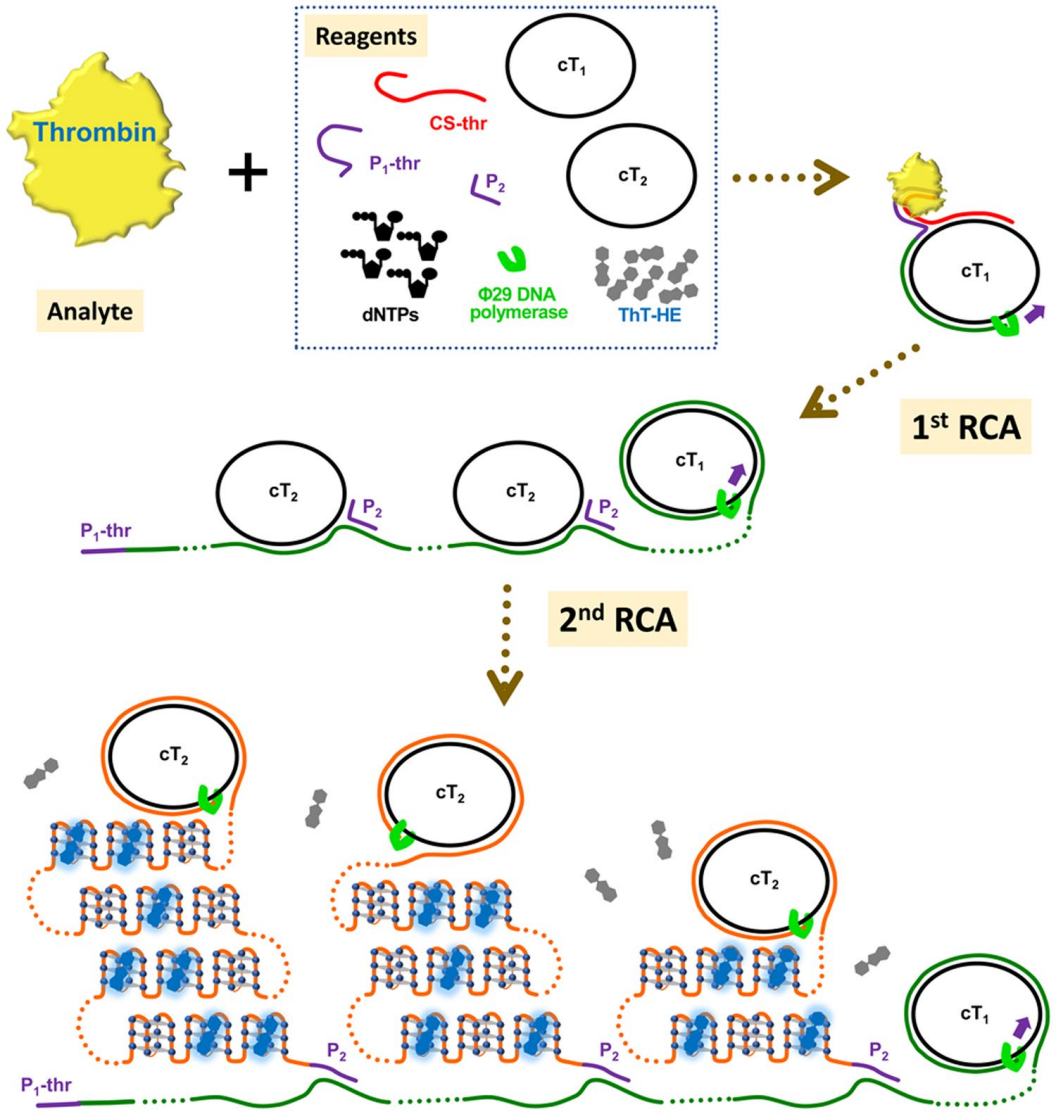
Schematic illustration of the one-tube, one-step light-up system for the visual detection of thrombin.

To this end, we employed split aptamers^14–18^ for target recognition and modified a φ29 DNA polymerase-catalyzed rolling circle amplification (RCA) system^19^ termed “signal amplification by ternary initiation complexes (SATIC)”^20^ as a platform of amplicon production. In principle, the formation of a four-membered initiation complex involving the target can trigger RCA, thereby generating polynucleotides containing tandemly tethered multiple G-quadruplexes that are specifically and fluorescently stained with a thioflavin T (ThT) derivative. The amplification can be chronologically monitored in a one-tube, one-step manner (Figs 1 and 2).

In the proposed system, the target (thrombin or streptomycin), capture strand (CS-thr or CS-str), and first primer (P_1_-thr or P_1_-str) associate on the circular template (cT_1_) to form a four-membered initiation complex to start RCA (the names and sequences of all oligonucleotides used in this study are listed in Table S1). The capture strands, CS-thr and CS-str, comprise an 11-mer and 17-mer split aptamer sequence (of thrombin and streptomycin, respectively) at the 3′-end, and a 20-mer common sequence at the 5′-end, which hybridizes to a part of cT_1_ (Table S1). The 3′-end of the strands is capped with monophosphates to prevent undesired extension during RCA. The primers, P_1_-thr and P_1_-str, contain a 10-mer and 21-mer split aptamer sequence at the 5′-end, respectively, and a 7-mer common sequence at the 3′-end. The 7-mer common sequence was optimized in length and sequence on the basis of nearest-neighbour thermodynamic parameters^21,22^. Specifically, without formation of the initiation complex, the primer (P_1_-thr or P_1_-str) cannot stably hybridize to the corresponding part on cT_1_, preventing extension with φ29 DNA polymerase. After the first RCA starts, the generated P_1_-thr- or P_1_-str-elongated strand attracts multiple circular templates (cT_2_) and, thereby, the second primer P_2_ is able to associate with cT_2_, which starts the second RCA to generate P_2_-elongated strands (Fig. 2). The amplicons (i.e., the P_2_-elongated strands) contain tandemly tethered multiple three-tiered G4s^23^, transcribed from cT_2_ that incorporate a 27-mer complementary sequence of c-Myc specifically stained with the ThT derivative (ThT-HE) (Figure S1)^24,25^. Overall, in principle, if the target is present, the test tubes are expected to emit fluorescence after adding the relevant reagents after maintenance of an isothermal temperature (37 °C).

## Results and Discussion

### Optimization of alkali metal ion concentration

Generally, the standard buffer for RCA does not contain Na^+^ or K^+^ because an increase in the salt concentration attenuates the strand displacement activity of φ29 DNA polymerase^26^; however, adjustment of alkali metal ion concentration was necessary. Indeed, RCA in our light-up system only worked at low concentrations up to 7.5 mM and 10 mM of Na^+^ and K^+^, respectively (Figure S2). Further, nether of the split aptamers exhibited their target-binding activities in the absence of those metal ions. As a result, RCA running and aptamer activities could simultaneously be sustained under the optimal condition of 10 mM of K^+^ (Figures S3 and S4).

### Verification of the four-membered initiation complex formation

**Figure 3:**
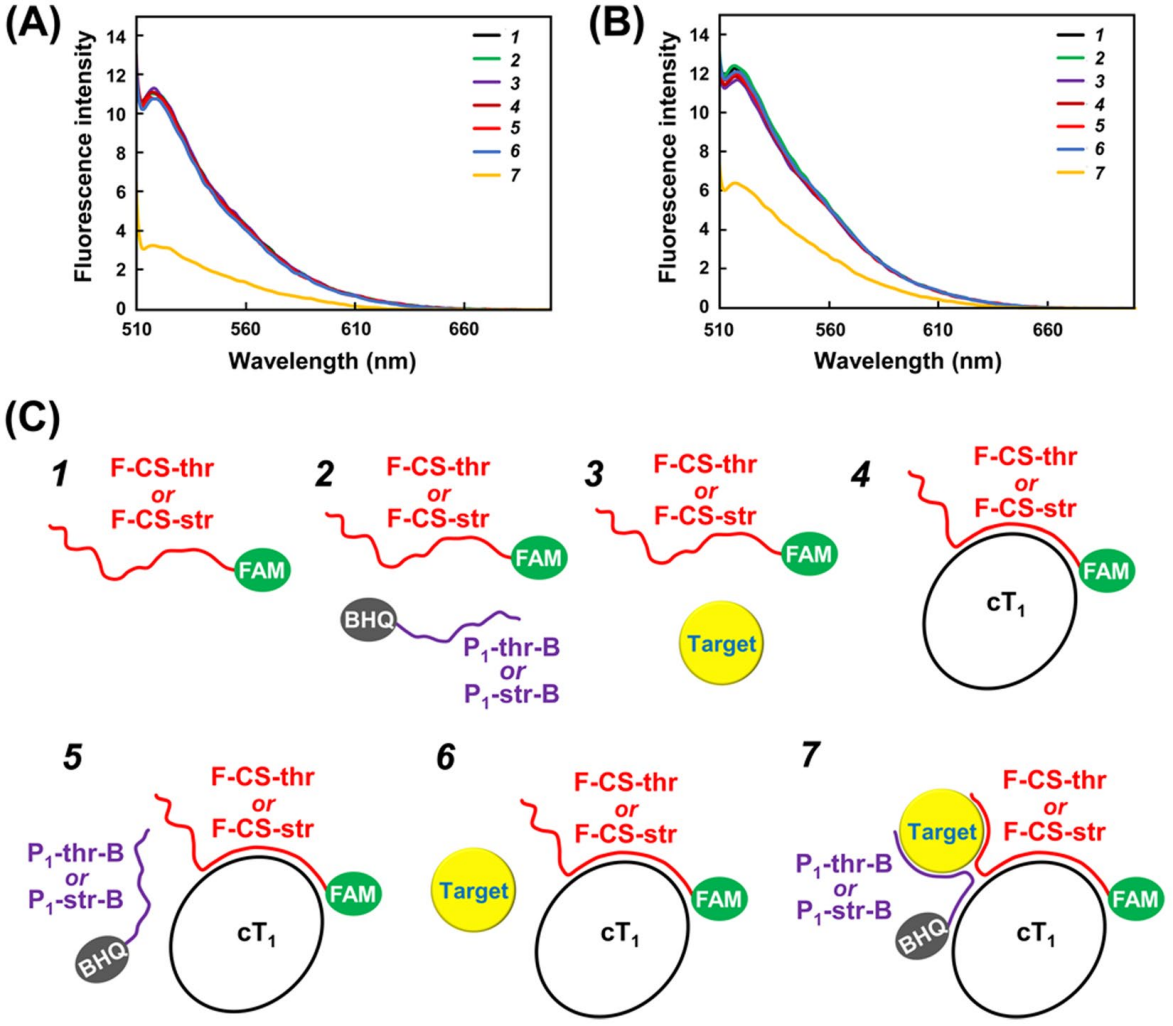
FRET quenching from the formation of a four-membered initiation complex (target, circular template, primer, and capture strands). The FRET experiments were conducted to verify the four-membered complex formation involving (**A**) thrombin and (**B**) streptomycin. F-CS-thr or F-CS-str (spectra *1*); F-CS-thr/P_1_-thr-B or F-CS-str/P_1_-str-B (spectra *2*); F-CS-thr/thrombin or F-CS-str/streptomycin (spectra *3*); cT_1_/F-CS-thr or cT_1_/F-CS-str (spectra *4*); cT_1_/F-CS-thr/P_1_-thr-B or cT_1_/F-CS-str/P_1_-str-B (spectra *5*); cT_1_/F-CS-thr/thrombin or cT_1_/F-CS-str/streptomycin (spectra *6*); cT_1_/F-CS-thr/P_1_-thr-B/thrombin or cT_1_/F-CS-str/P_1_-str-B/streptomycin (spectra *7*) as illustrated in (**C**). Only spectra *7* showed efficient FRET quenching from complex formation.

To verify the four-membered initiation complex formation on cT_1_ (Fig. 1), fluorescence resonance energy transfer (FRET) experiments were conducted using 6-carboxyfluorescein (5′-FAM)-labelled capture strands (F-CS-thr or F-CS-str) and 3′-BHQ (Black Hole Quencher 1)-labelled primers (P_1_-thr-B or P_1_-str-B) with or without the targets (thrombin or streptomycin). As shown in Fig. 3, efficient FRET quenching was observed only when all four members (cT_1_/F-CS-thr/P_1_-thr-B/thrombin or cT_1_/F-CS-str/P_1_-str-B/streptomycin) were present. These results indicate that complex formation can trigger the start of RCA and enable specific target detection using the SATIC methodology (Fig. 2).

### Visual and quantitative detection of a protein target

**Figure 4:**
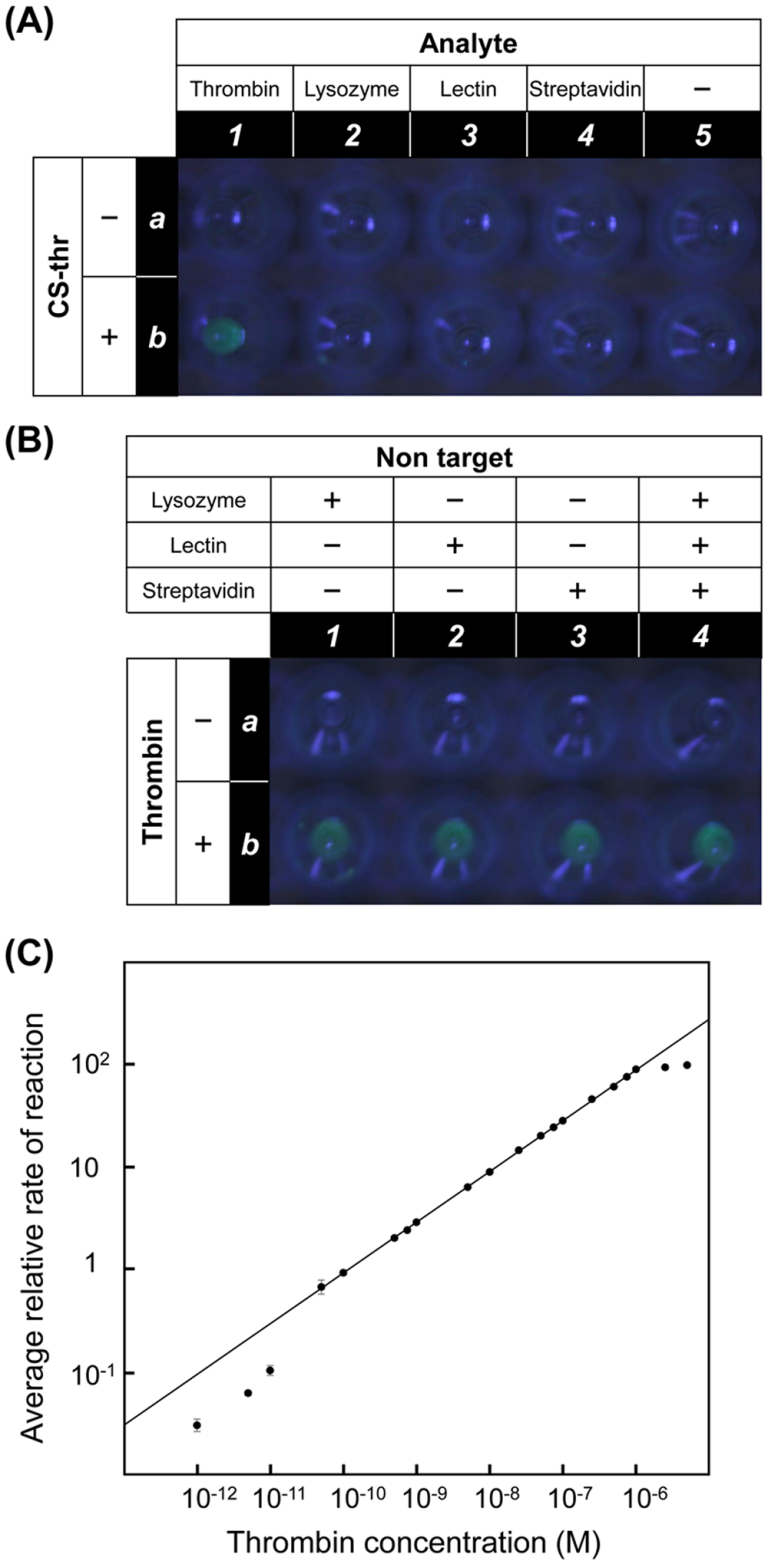
The specific light-up system for protein target detection: photographs of the aliquots containing ThT-HE (5 μM) captured under visible light irradiation at 410 nm. Each reaction mixture was incubated at 37 °C for 2 h. (**A**) The aliquots in rows a and b are reaction mixtures without and with the capture strand CS-thr, respectively. Similarly, the aliquots in columns 1, 2, 3, 4, and 5 contain an analyte (1.0 nM) as described. (**B**) The aliquots in rows a and b are reaction mixtures without and with the target thrombin (1.0 nM), respectively. Similarly, the aliquots in columns 1, 2, 3, and 4 contain analyte(s) (1.0 nM each) as described, where + and − indicate presence and absence, respectively. Real-time quantitative analysis by the light-up system (**C**): linear calibration curve for target quantitation in the range from 0.050 to 1000 nM (R^2^ = 0.9998). Error bars reflect three separate measurements.

To demonstrate the feasibility of the envisioned light-up system, we first examined detection of human thrombin as a protein target. Measurement of thrombin generation, e.g., by a thrombin generation assay, has recently gained renewed attention in the clinical areas of thrombosis and haemostasis due to the increasing demand for the development of simple haemostatic ability diagnoses for individual patients against anti-coagulant drugs^27^. As shown in Fig. 4A, thrombin was specifically detected with greenish blue fluorescence (λ_ex_ = 410 nm) and no emission was observed in test tubes containing non-targets such as lysozyme, lectin, streptavidin, and in the negative-control tube without any proteins. Without the capture strand (CS-thr), no emission was observed in the presence of thrombin, indicating that the two split aptamer strands are consistently associated by thrombin on the circular template to form the initiation complex for signal amplification by RCA. Furthermore, thrombin was clearly visualized in the presence of three non-target proteins (Fig. 4B). Next, we attempted quantitative detection of thrombin using a CFX96 real-time PCR detection system (Bio-Rad Laboratories, Inc., CA, USA). The average relative rate of reaction for each target concentration (0–5000 nM) was obtained from the increase in fluorescence intensity per unit time (Figure S5A). As shown in Fig. 4C, the logarithm of the average relative rate of reaction was linearly proportional to the logarithm of the thrombin concentration in the range of 0.050–1000 nM. Currently, the detection limit of the system is between 10 and 50 pM (i.e., at most 50 pM) for thrombin. Notably, no increase in fluorescence intensity was observed in the absence of the target, even 100 min after the start of monitoring (Figure S5). This result indicates that RCA initiation in the light-up system can be strictly regulated by the presence of the analyte.

The 14-mer and 13-mer sequences of the thrombin-binding split aptamer imitate a thrombin-binding aptamer (TBA) consisting of a 29-mer deoxyribonucleotide (DNA) that forms a hairpin loop with a two-tiered G4^28–32^. While the 29-mer TBA is well known as a high-affinity aptamer with a K_d_ value of 0.5 nM, the binding affinity of the split aptamer, which was divided in the middle of the G4 moiety, may be slightly degraded but was substantially retained^14^. Thus, as expected, our experimental results prove that the split aptamer can act as a key element for the switching machinery.

In many cases, crude biological samples contain nucleases, which may affect the outcome of target analyses using the present methodology. In φ29 buffers containing 10%, 30%, and 60% v/v human serum, 26-mer single-stranded oligodeoxyribonucleotides (T_26_) were greatly degraded within 2 h at 37 °C (Figure S6). From these conditions, 10% v/v human serum exhibited the strongest nuclease activity. Therefore, we attempted the specific light-up of thrombin under such conditions and confirmed that SATIC was effective, although the fluorescence intensity was somewhat decreased (Figure S7), while using a modified-type primer, P_1_-thr-PS, instead of a natural-type primer, P_1_-thr (Table S1). P_1_-thr-PS remained substantially intact in 10% v/v human serum for 2 h at 37 °C while P_1_-thr was almost entirely digested (Figure S8). These results indicate that further chemical modifications of the nucleotide components used for SATIC will yield better detection systems^29,33–36^.

### Visual and quantitative detectio.n of a small molecular target

**Figure 5:**
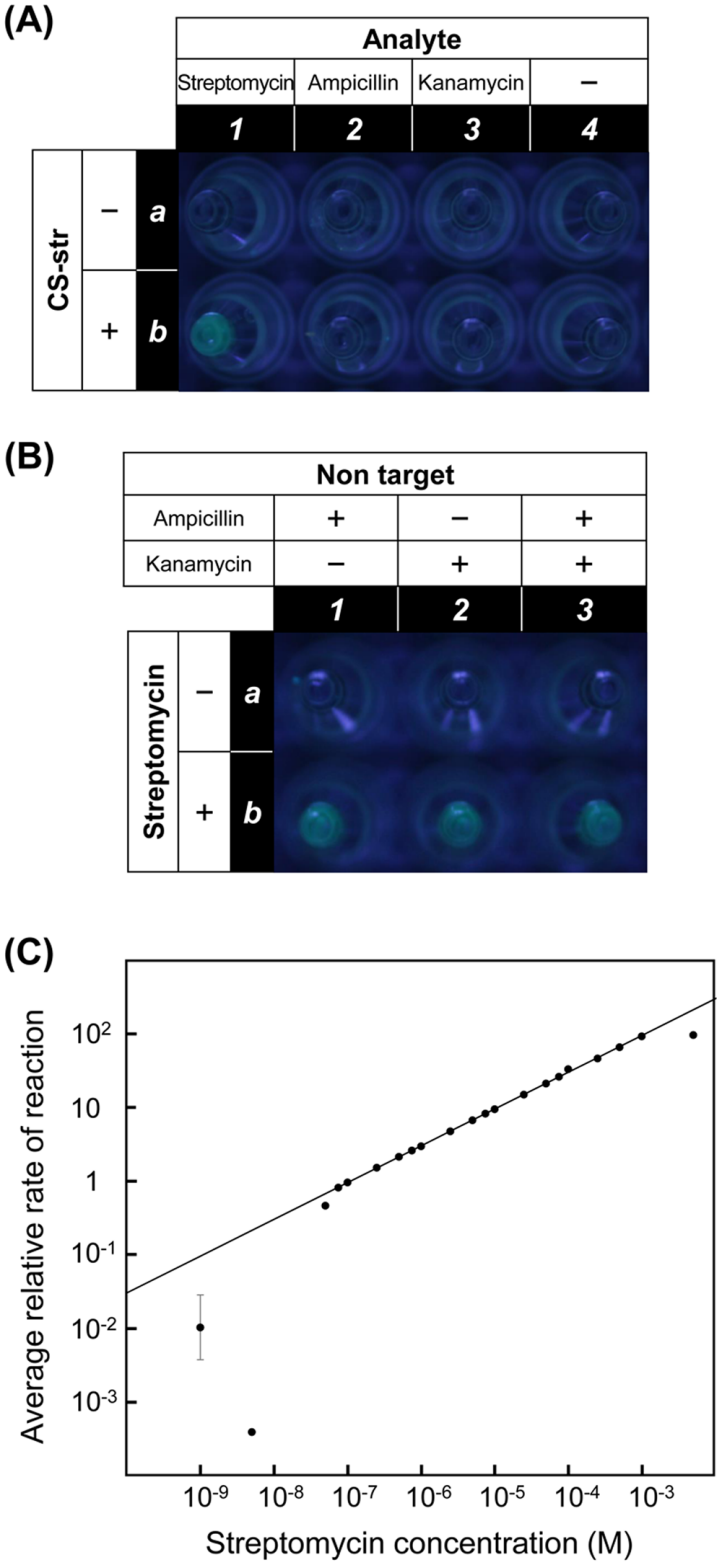
The specific light-up system for small molecular target detection: photographs of the aliquots containing ThT-HE (5 μM) captured under visible light irradiation at 410 nm. Each reaction mixture was incubated at 37 °C for 2 h. (**A**) The aliquots in rows a and b are reaction mixtures without and with the capture strand CS-str, respectively. Similarly, the aliquots in columns 1, 2, 3, and 4 contain an analyte (0.10 μM) as described. (**B**) The aliquots in rows a and b are reaction mixtures without and with the target streptomycin (0.10 μM), respectively. Similarly, the aliquots in columns 1, 2, and 3 contain analyte(s) (0.10 μM each) as described, where + and − indicate presence and absence, respectively. Real-time quantitative analysis by the light-up system (**C**) linear calibration curve for target quantitation in the range from 0.075 to 1000 μM (R^2^ = 0.9997) for streptomycin. Error bars reflect three separate measurements.

To demonstrate the versatility of our light-up system, we examined the specific detection of streptomycin, for which the maximum residual levels of certain animal origin products have been set and strictly monitored for food hygiene, such as small molecular targets^37^. The streptomycin-binding split aptamer, whose 46-mer mother aptamer is known to exhibit an apparent K_d_ value of 1 μM^38^, consists of 18-mer and 22-mer ribonucleotide (RNA) fragments. The light-up system for streptomycin detection was simply designed by replacing the sequences of the thrombin-binding split aptamer with those of the streptomycin-binding split aptamer. Namely, CS-str and P_1_-str as DNA/RNA chimeric oligomers were synthesized and used for the experiments instead of CS-thr and P_1_-thr. As with the thrombin light-up system, the target streptomycin was successfully distinguished from other coexisting metabolites such as ampicillin and kanamycin (Fig. 5A,B). Furthermore, streptomycin was quantitatively detected in the concentration range of 0.075–1000 μM when the detection limit of the system was between 0.050 and 0.075 μM (i.e., at most 0.075 μM) (Fig. 5C), which is higher than the range of thrombin attributable to the difference between the target-binding affinities of the two split aptamers.

## Conclusions

We have constructed a one-tube, one-step light-up system for non-nucleic acid targets using isothermal nucleic acid amplification techniques. Furthermore, we demonstrated quantitative performance over a wide dynamic range and sequence compatibility with nucleotide type (i.e., DNA and RNA) in the aptamer parts, enabling diverse biomarker detection. Although several methods for such bioanalyses using RCA^39–45^ or loop-mediated isothermal amplification (LAMP)^46,47^ have been devised, the present method substantiated quantitative measurements without any operations such as annealing, washing, or transferring of samples^48–51^. Furthermore, introduction of chemical modifications into the nucleotide components enhanced nuclease resistance, thus offering the possibility for practical application, such as the analyses of crude biological samples. While split aptamers were employed in this study for initiating RCA, the mechanisms of riboswitches^52–56^, which are known to control gene expressions by their conformational change caused by metabolite binding to their aptamer moiety^35,57–61^, will also be applicable to this system. Thus, our concept of assembly to start amplifications will readily expand the kinds of targets that can be used for measurement and will further facilitate simple diagnoses and tests with improved accuracy.

## Electronic supplementary material

Supplementary Information
