# Supplementary material for: Specific Light-Up System for Protein and Metabolite Targets Triggered by Initiation Complex Formation
Source: Sci Rep. 2017 Nov 9;7:15191. doi: 10.1038/s41598-017-15697-8 (PMC5680199; doi:10.1038/s41598-017-15697-8)
Supplement: Supplementary file 1 — Supplementary Information [file 41598_2017_15697_MOESM1_ESM.pdf]

Supplementary Information

Specific Light-Up System for Protein and Metabolite Targets Triggered by Initiation Complex Formation

Hiroto Fujita[1], Yuka Kataoka[1], Remi Nagano[1], Yasuyo Nakajima[2], Masanobu Yamada[2], Naoki Sugimoto[3,4], and Masayasu Kuwahara[*,1]

[1] Graduate School of Science and Technology, Gunma University, 1-5-1 Tenjin-cho, Kiryu, Gunma 376-8515, Japan

[2] Department of Internal Medicine, Division of Endocrinology and Metabolism, Graduate School of Medicine, Gunma University, 3-39-15 Showa-machi, Maebashi 371-8511, Japan

[3] Frontier Institute for Biomolecular Engineering Research (FIBER), Konan University, 7-1-20 Minatojima-minamimachi, Kobe 650-0047, Japan

[4] Graduate School of Frontiers of Innovative Research in Science and Technology (FIRST), Konan University, 7-1-20 Minatojima-minamimachi, Kobe 650-0047, Japan

Correspondence:

Professor M. Kuwahara, Gunma University (E-mail: mkuwa@gunma-u.ac.jp)

Materials

ThT-HE was synthesized according to our previously reported method[1] (Figure S1). Disodium hydrogen phosphate (Na_2HPO_4), lysozyme, magnesium chloride hexahydrate ($MgCl_2$•$6H_2O$), kanamycin, potassium chloride (KCl), sodium chloride (NaCl), sodium dihydrogen phosphate dihydrate (NaH_2PO_4•$2H_2O$), streptavidin, and streptomycin were purchased from Wako Pure Chemical Industries, Ltd. (Osaka, Japan). Human thrombin was purchased from Sigma-Aldrich, Inc. (MO, USA). Human serum was purchased from Merck, Ltd. (Tokyo, Japan). ϕ29 DNA polymerase was purchased from New England Biolabs Japan, Inc. (Tokyo, Japan). The four dNTPs (dATP, dGTP, dCTP, and TTP) and ampicillin were purchased from Roche Diagnostics K.K. (Tokyo, Japan). SPECTROZYME® TH was purchased from Sekisui Diagnostics, LLC (MA, USA). The ODNs (T1, T2, CS-thr, CS-str, P0, P1-thr, P1-str, P2, F-CS-thr, F-CS-str, P_1-thr-B, P1-str-B, P1-thr-PS and T_{26}) were purchased from Japan Bio Services Co. Ltd. (Saitama, Japan) or GeneDesign Inc. (Osaka, Japan), and their sequences are given in Table S1. Buffers and solutions, e.g., 5× PBS (50 mM sodium phosphate, 690 mM NaCl, 13.5 mM KCl, 12.5 mM $MgCl_2$, pH 7.4) was prepared using ultrapure water obtained using a CPW-100 ultrapure water system (Advantec Co. Ltd., Tokyo, Japan).

Effects of alkali metal ion concentration on RCA

Alkali metal ions (Na^+ or K^+; 2µL) at 9 different concentrations (0, 10, 25, 50, 75, 100, 250, 500, and 1000 mM) were added to a mixture (18 µL) that was comprised of primer P_0 (5′-TCT TCG ACA ACA ATT ATC ACT T-3′; 480 nM, 2 µL), circular DNA template cT_2 (400 nM, 2 µL) (which could be obtained from the corresponding liner form in compliance with a previous report[2]), dNTP mix (dATP, dCTP, dGTP, and TTP; 10 mM each, 2 µL), ϕ29 DNA polymerase (1 U/µL, 2 µL), 10× ϕ29 buffer (2 µL), BSA solution (10 mg/mL, 0.2 µL), and ultrapure water (7.8 µL). Each reaction mixture (20 µL) was incubated at 37 °C for 2 h, quenched with 5× PBS (5 µL), and mixed with 4 µL of ThT-HE (30 µM) solution in 1× PBS.

The solutions were incubated at room temperature for 30 min and photographs were taken using a EOS KISS X70 digital camera (Canon Inc., Tokyo, Japan) under visible-light irradiation at 410 nm

using an 8 mm super violet LED OSSV8131A (OptoSupply Limited, Hong Kong, China) through a <460 nm Fuji Filter Optical SC-46 cut-off filter (Fujifilm Corporation, Tokyo, Japan) (Figure S2).

Optimization of alkali metal ion concentration for thrombin detection

Target protein (thrombin; 10 nM, 2µL) was added to a mixture (18 µL) that was comprised of the capture strand CS-thr (120nM, 2µL), primer P1-thr (120 nM, 2 µL), primer P2 (480 nM, 2 µL), circular DNA template cT1 (200 nM, 1 µL), circular DNA template cT2 (800 nM, 1 µL), dNTP mix (dATP, dCTP, dGTP, and TTP; 10 mM each, 2 µL), ϕ29 DNA polymerase (1 U/µL, 2 µL), 10× ϕ29 buffer (2 µL), BSA solution (10 mg/mL, 0.2 µL), potassium chloride (1 µL) at 5 different concentrations (0, 40, 200, 500, and 1,000 mM) and ultrapure water (2.8 µL). Each reaction mixture (20 µL) was incubated at 37 °C for 2 h, quenched with 5× PBS (5 µL), and mixed with 4 µL of ThT-HE (30 µM) solution in 1× PBS. The solutions were then incubated at room temperature for 30 min and photographed in the same way as described above (Figure S3A).

Potassium ion dependency on the detection reaction was more precisely analyzed (Figure S4AB). Potassium ion (K^+; 3µL) at 11 different concentrations (0, 40, 80, 120, 160, 200, 240, 280, 320, 360, and 400 mM) was added to a mixture (57 µL) comprising capture strand CS-thr (240 nM, 3 µL), primer P_1-thr (240 nM, 3 µL), primer P2 (480 nM, 6 µL), circular DNA template cT_1 (100 nM, 6 µL), circular DNA template cT_2 (400 nM, 6 µL), dNTP mix (dATP, dCTP, dGTP, and TTP; 10 mM each, 6 µL), ϕ29 DNA polymerase (1 U/µL, 6 µL), 10× ϕ29 buffer (6 µL), BSA solution (10 mg/mL, 0.6 µL), target protein (thrombin; 10 nM, 6µL), and ultrapure water (8.4 µL). Each reaction mixture (60 µL) was incubated at 37 °C for 2 h, quenched with 5× PBS (15 µL), and mixed with a 12.5 µL of ThT-HE (30 µM) solution in 1× PBS. The emission spectra of the dyes were obtained by excitation at 415 nm and fluorescence monitoring between 450 and 650 nm with an LS-55 spectrofluorophotometer (PerkinElmer Japan Co., Ltd., Kanagawa, Japan).

Optimization of alkali metal ion concentration for streptomycin detection

Target metabolite (streptomycin; 1.0 µM, 2 µL) was added to a mixture (18 µL) that was comprised of capture strand CS-str (120 nM, 2 µL), primer P_1-str (120 nM, 2 µL), primer P_2 (480 nM, 2 µL),

circular DNA template cT_1 (200 nM, 1 µL), circular DNA template cT_2 (800 nM, 1 µL), dNTP mix (dATP, dCTP, dGTP, and TTP; 10 mM each, 2 µL), ϕ29 DNA polymerase (1 U/µL, 2 µL), 10× ϕ29 buffer (2 µL), BSA solution (10 mg/mL, 0.2 µL), potassium chloride (1 µL) at 5 different concentrations (0, 40, 200, 500, and 1000 mM) and ultrapure water (2.8 µL). Each reaction mixture (20 µL) was incubated at 37 °C for 2 h, quenched with 5× PBS (5 µL), and mixed with 4 µL of ThT-HE (30 µM) solution in 1× PBS. The solutions were then incubated at room temperature for 30 min and photographed in the same way as described above (Figure S3B).

Potassium ion dependency on the detection reaction was more precisely analyzed (Figure S4CD). Potassium ion (K^+; 3µL) at 11 different concentrations (0, 40, 80, 120, 160, 200, 240, 280, 320, 360, and 400 mM) was added to a mixture (57 µL) comprising capture strand CS-str (240 nM, 3 µL), primer P_1-str (240 nM, 3 µL), primer P2 (480 nM, 6 µL), circular DNA template cT_1 (100 nM, 6 µL), circular DNA template cT_2 (400 nM, 6 µL), dNTP mix (dATP, dCTP, dGTP, and TTP; 10 mM each, 6 µL), ϕ29 DNA polymerase (1 U/µL, 6 µL), 10× ϕ29 buffer (6 µL), BSA solution (10 mg/mL, 0.6 µL), target metabolite (streptomycin; 1.0 µM, 6µL), and ultrapure water (8.4 µL). Each reaction mixture (60 µL) was incubated at 37 °C for 2 h, quenched with 5× PBS (15 µL), and mixed with a 12.5 µL of ThT-HE (30 µM) solution in 1× PBS. The emission spectra of the dyes were obtained by excitation at 415 nm and fluorescence monitoring between 450 and 650 nm with an LS-55 spectrofluorophotometer.

Correlation line and detection limits

The real-time quantitative analyses of thrombin and streptomycin in the abovementioned 21 and 22 different concentrations were conducted using a CFX96 real-time PCR detection system, respectively (Figures 4C, 5C, and S5). Respective relative rates of reaction (A) were obtained by fitting the time course data of fluorescence intensity from 0 to 100 min with equation 1, where I is the fluorescence intensity, t is the reaction time, A is the relative rate of reaction, and B is the y-intercept.

$$\mathrm{I} = \mathrm{A}t + \mathrm{B} \qquad eq. 1$$

Three independent measurements (n = 3) were performed at a single concentration for all A–value determinations. Standard deviations (m) were calculated using equation 2, where Ā is the average of those measurements (A_1, A_2, and A_3).

$$m = \sqrt{\frac{(A_1 - \bar{A})^2 + (A_2 - \bar{A})^2 + \cdots + (A_n - \bar{A})^2}{n}} \qquad eq.2$$

The logarithmic values of Ā (*log* Ā) with error bars that show the standard deviations (σ) were plotted versus the logarithm of target concentrations, as shown in Figures 4C and 5C. The standard deviations (σ) were calculated using equation 3.

$$\sigma = \frac{1}{ln10} \cdot \frac{m}{\bar{A}} \qquad eq.3$$

The correlation lines, equations 4 for thrombin and 5 for streptomycin, could be plotted between 0.050 and 1000 nM and 0.075 and 1000 μM with a high correlation coefficient (R^2 = 0.9998 and 0.9997), respectively.

$$y = 0.497x + 4.93 \qquad eq.4$$

$$y = 0.500x + 3.49 \qquad eq.5$$

The present system achieved detection limits of ca. 50 pM for thrombin and ca. 75 nM for streptomycin at the highest estimate, respectively. Namely, the present system provided signal intensities with small σ values at low target concentrations: *log* Ā ± σ was equal to –0.990 ± 0.050 and –0.174 ±0.071 at thrombin concentrations of 10 and 50 pM, respectively; while *log* Ā ± σ was equal to –0.329 ± 0.018 and –0.082 ± 0.012 at streptomycin concentrations of 50 and 75 nM, respectively.

Target detection in human serum

Target protein (thrombin; 10 nM, 2μL) was added to a mixture (18 μL) containing CS-thr (240 nM, 1 μL), P_1-thr or P_1-thr-PS (240 nM, 1 μL), P_2 (480 nM, 2 μL), cT_1 (100 nM, 2 μL), cT_2 (400 nM, 2 μL), dNTP mix (dATP, dCTP, dGTP, and TTP; 10 mM each, 2 μL), ϕ29 DNA polymerase (1 U/μL, 2 μL), 10× ϕ29 buffer (2 μL), BSA solution (10 mg/mL, 0.2 μL), potassium chloride (200 mM, 1 μL), and ultrapure water (0.8 μL) in addition to human serum (2 μL) or additional ultrapure water (2 μL) for the control. Each reaction mixture (20 μL) was incubated at 37 °C for 2 h, quenched with 5× PBS

(5 µL), and mixed with 4 µL of ThT-HE (30 µM) solution in 1× PBS. The solutions were then incubated at room temperature for 30 min and imaged in the same manner described above (Figure S7).

Stabilities of the natural/modified oligonucleotides in human serum

Reactions (10 µL reaction volume) containing 1 µL of ϕ29 buffer (10×) and 1 µL of oligonucleotide (T_{26}, P_1-thr, or P_1-thr-PS (4 µM)) were performed in 10% v/v human serum for all ODNs. In addition, experiments were conducted in 30% and 60% v/v human serum for T_{26}. All reactions were incubated at 37 °C for 2 h. The reaction products were resolved by denaturing PAGE, and gel images were recorded with excitation of the 5′-labeled fluorophore at 488 nm (Figure S6A for degradation of T_{26}) or SYBR® Gold at 488 (Figure S8A for degradation of P_1-thr and P_1-thr-PS), and the band intensities were quantified by the Quantity One software (Figures S6B and S8B). The decay curves of intact ODN were fitted from band intensities at appropriate intervals of reaction time (0, 10, 30, 60, and 120 min) by the least squares method using the OriginProver. 8.1 program.

Measurement of thrombin content in human serum

For the experiment to measure thrombin content in human serum, four tests were performed using 2 nM thrombin in ϕ29 buffer (1×), 20 nM thrombin in ϕ29 buffer (1×), 20% human serum in ϕ29 buffer (1×), and ϕ29 buffer (1×) for the blank. For each test, 40 µL of solution was mixed with an equal volume of ϕ29 buffer (1×) containing SPECTROZYME® TH (1 mM) (each solution was warmed to 37 °C prior to mixing). Then, the four test samples were incubated at 37 °C and the degradation was monitored for 2000 s using a UV-2700 spectrophotometer (Shimadzu corporation, Tokyo, Japan). The changes in absorbance at 405 nm were recorded at 2 s intervals (Figure S9).

REFERENCES

1. Kataoka, Y. *et al.* Minimal thioflavin T modifications improve visual discrimination of guanine-quadruplex topologies and alter compound-induced topological structures. *Anal. Chem.* **86**, 12078–12084 (2014).

2. Fujita, H., Kataoka, Y., Tobita, S., Kuwahara, M. & Sugimoto, N. Novel One-Tube-One-Step

Real-Time Methodology for Rapid Transcriptomic Biomarker Detection: Signal Amplification by Ternary Initiation Complexes. *Anal. Chem.* **88**, 7137–7144 (2016).

3. Fujita, H. *et al.* Polymerase-mediated high-density incorporation of amphiphilic functionalities into DNA: Enhancement of nuclease resistance and stability in human serum. *Bioorg. Med. Chem. Lett.* **25,** 333–336 (2015).

Table S1. Sequences of circular templates, capture strands, and primers

Oligonucleotides	Sequence[a]
cT_1	CCCAAAAAAGGAGCTTGAGGTTCTCCTTTAAAAAGAAGCTGTTGTATTGTTGTCGAAGAAGAAAAGT[b]
cT_2	CCCAACCCTACCCACCCTCAAGAAAAAAAAAGTGATAATTGTTGTCGAAGAAGAAAAAAAAATT[b]
CS-thr	AAAGGAGAACCTCAAGCTCCTTTGGTTGGTG
CS-str	AAAGGAGAACCTCAAGCTCCggcaccacggucggauc[c]
P_1-thr	TGGTTGGAAATTTTTTG
P_1-str	gaucgcauuuggacuucugccTTTTTTG[c]
P_2	GAAGCTGTTGTTATCACT
F-CS-thr	FAM-AAAGGAGAACCTCAAGCTCCTTTGGTTGGTG
F-CS-str	FAM-AAAGGAGAACCTCAAGCTCCggcaccacggucggauc[c]
P_1-thr-B	TGGTTGGAAATTTTTTG-BHQ
P_1-str-B	gaucgcauuuggacuucugccTTTTTTG-BHQ[c]
P_1-thr-PS	TGGTTGGAAATTTTT($_{ps}$T)($_{ps}$G)[d]
T_{26}	FAM-TTTTTTTTTTTTTTTTTTTTTTTTTT

[a]Sequences are aligned in the 5′ to 3′ direction. [b]Strands were cyclized at both the 5′- and 3′-ends via ligation into circular template strands. [c]Lower case letters indicate ribonucleotides. [d](*ps*T) = thymidine 5′-*O*-phosphorothioate and (*ps*G) = 2′-deoxyguanoasine 5′-*O*-phosphorothioate.

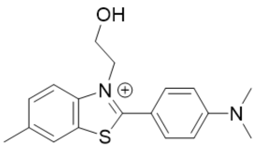

ThT-HE

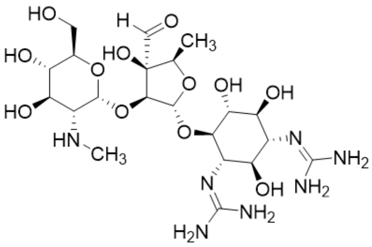

Streptomycin

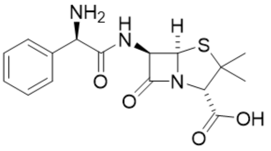

Ampicillin

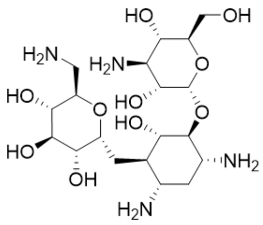

Kanamycin

Figure S1. Chemical structures of the compounds used in this study.

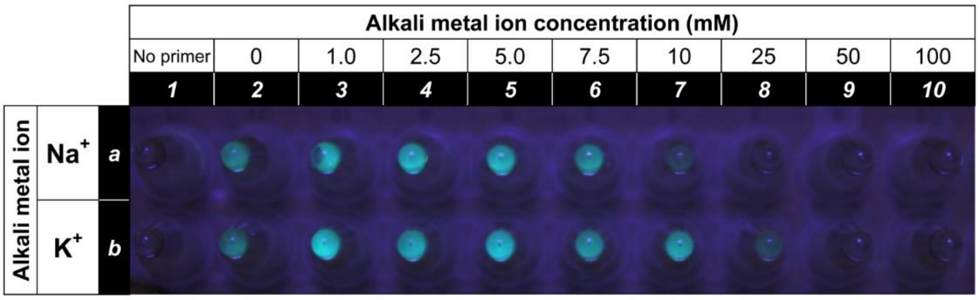

Figure S2. Effects of alkali metal ion concentration on RCA: a photograph of the aliquots containing ThT-HE (5 μM) captured under visible light irradiation at 410 nm. Each reaction mixture was incubated at 37 °C for 2 h. The aliquots in rows a and b are reaction mixtures with Na^+ and K^+, respectively. Similarly, the aliquots in column 1 did not contain primer P_0, and the aliquots in columns 2, 3, 4, 5, 6, 7, 8, 9, and 10 contain P_0 in addition to 0, 1.0, 2.5, 5.0, 7.5. 10, 25, 50, and 100 mM of the indicated metal ion, respectively.

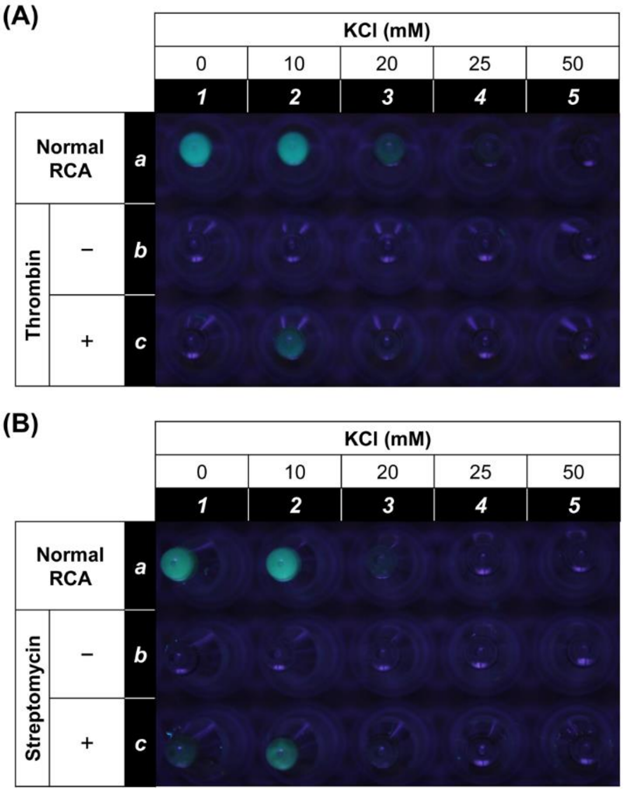

Figure S3. Optimization of alkali metal ion concentration (A) for thrombin detection and (B) for streptomycin detection. Photographs of the aliquots containing ThT-HE (5 µM) captured under visible light irradiation at 410 nm. Each reaction mixture was incubated at 37 °C for 2 h. The aliquots in rows a, b, and c are reaction mixtures of normal RCA using primer P_0, and those without and with the target as described, respectively. Similarly, the aliquots in the columns labeled 1, 2, 3, 4, and 5 contain 0, 10, 20, 25, and 50 mM of K^+, respectively.

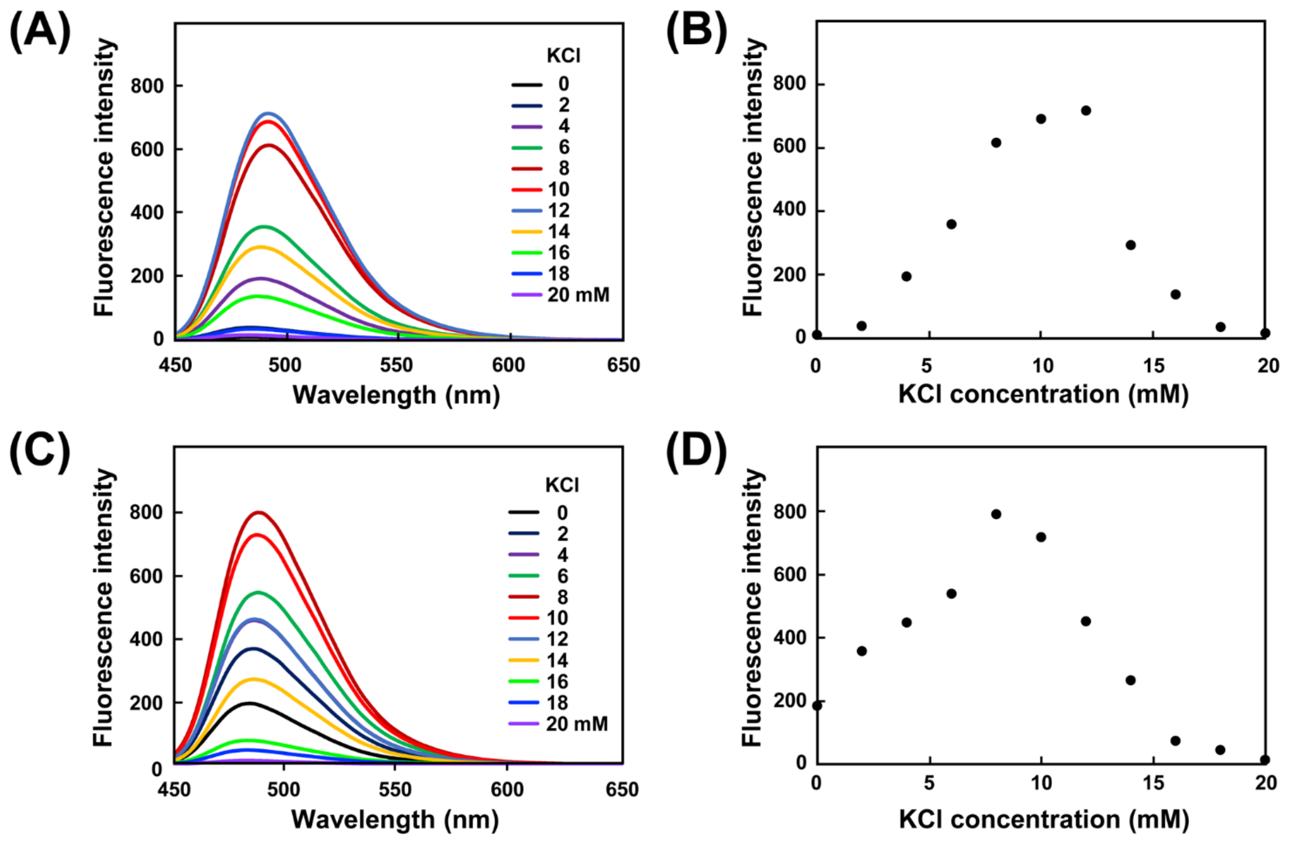

Figure S4. Optimization of alkali metal ion concentration (A, B) for thrombin detection and (C, D) for streptomycin detection. Fluorescence spectra (A, C) of the aliquots containing ThT-HE (5 μM) measured under excitation at 410 nm. Each reaction mixture was incubated at 37 °C for 2 h. Maximum fluorescence intensities (B, D) at different K^+ concentrations (0–20 mM).

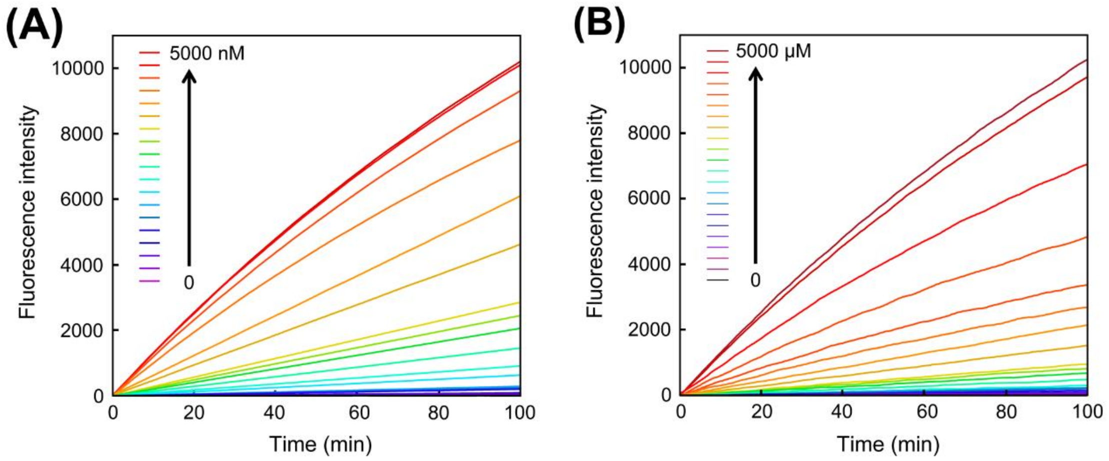

Figure S5. Real-time quantitative analysis by the light-up system: time course of fluorescence intensity of ThT-HE (5 μM) with different concentrations (A) of the target protein, thrombin (0–5000 nM), and (B) of the target metabolite, streptomycin (0–5000 μM).

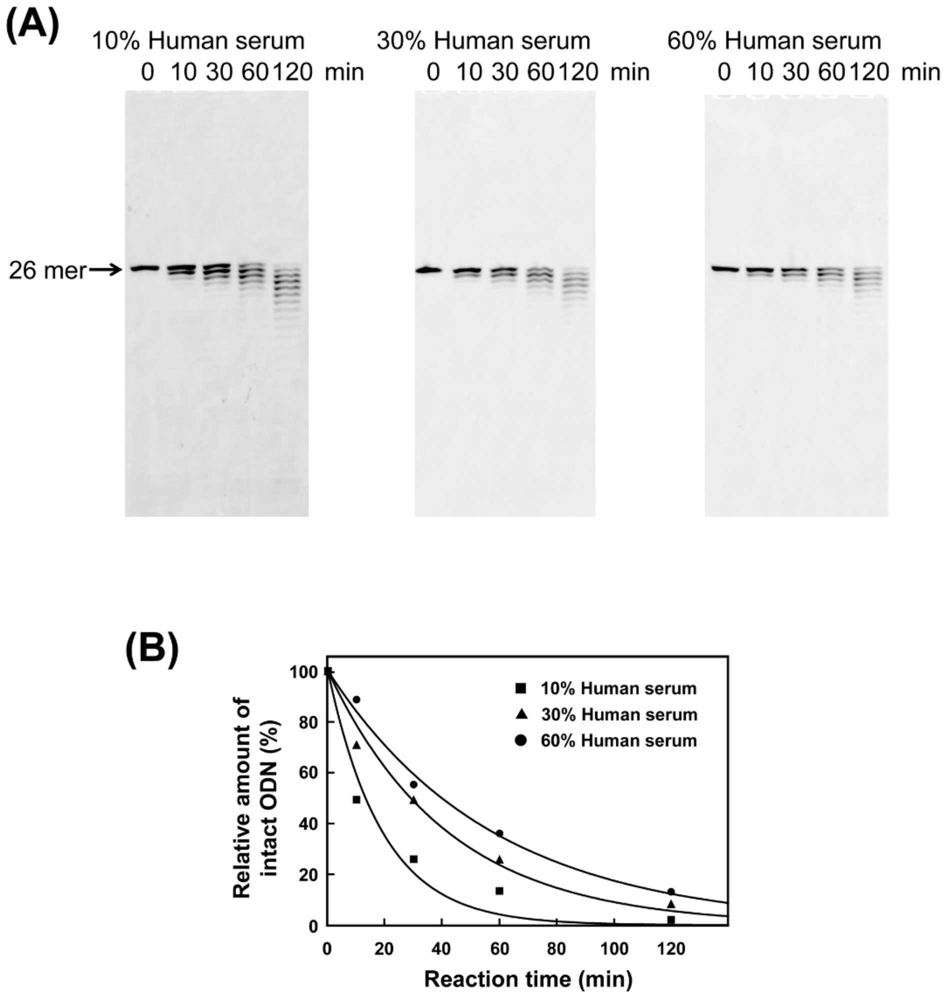

Figure S6. (A) Degradation of 26-mer ODN (T_{26}) in 10%, 30%, and 60% v/v human serum analyzed by gel electrophoresis. The reaction mixtures at 0, 10, 30, 60, and 120 min were loaded into the wells as indicated. (B) Time-dependent degradation of T_{26} in human serum: 10% (square), 30% (triangle), and 60% human serum (circle). The reaction time (in minutes) is shown on the x-axis and the relative amount of intact ODN (%) is shown on the y-axis. Initial quantity of the ODN was assumed to be at 100% in each reaction mixture.

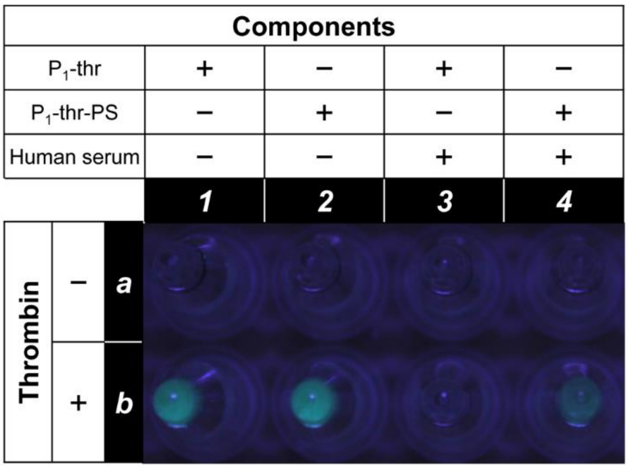

Figure S7. Thrombin detection in human serum. Photographs of the tests containing ThT-HE (5 µM) captured under visible light irradiation at 410 nm. Each reaction mixture was incubated at 37 °C for 2 h. The tests in rows a and b are reaction mixtures without and with target (thrombin), respectively. The tests in columns 1 and 3 contain P_1-thr as a natural type DNA primer while those in columns 2 and 4 contain P_1-thr-PS as a modified type DNA primer; furthermore, columns 3 and 4 contain 10% v/v human serum.

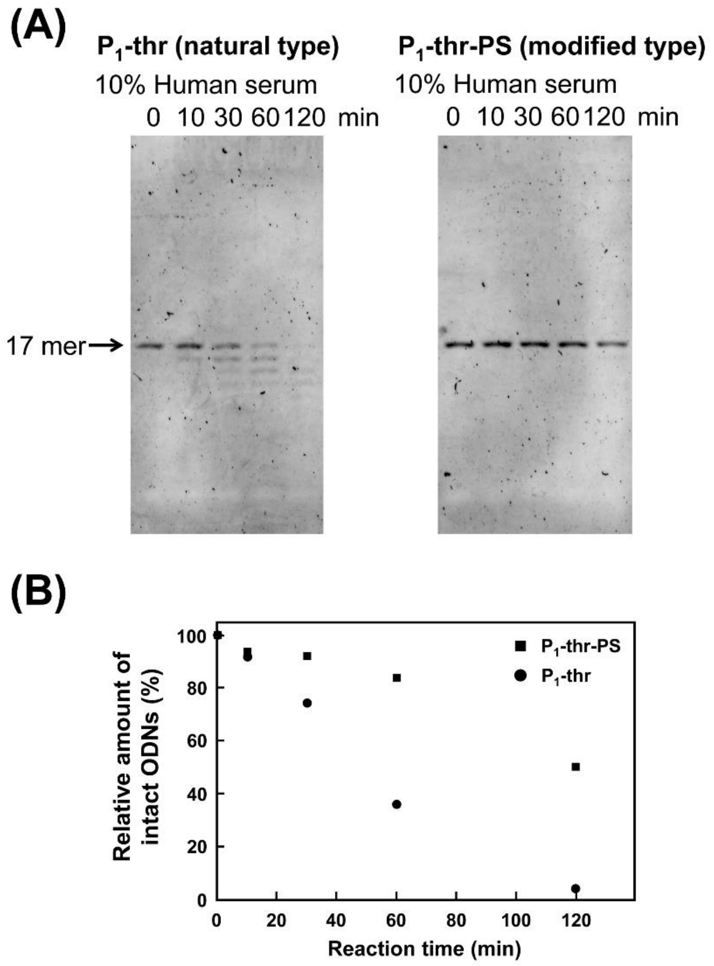

Figure S8. (A) Degradation of P_1-thr and P_1-thr-PS in 10% v/v human serum analyzed by gel electrophoresis. The reaction mixtures at 0, 10, 30, 60, and 120 min were loaded into the wells as indicated. (B) Time-dependent degradation of ODNs in 10% v/v human serum: P_1-thr-PS (square) and P_1-thr (circle). The reaction time (in minutes) is shown on the x-axis and the relative amount of intact ODNs (%) is shown on the y-axis. Initial quantities of the ODNs were assumed to be at 100% in each reaction mixture.

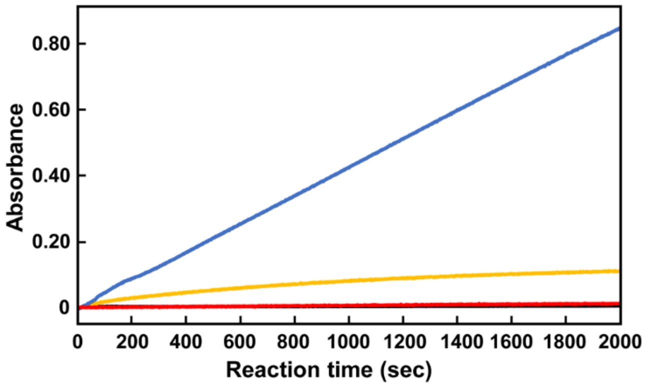

Figure S9. Analysis of thrombin activity in human serum using SPECTROZYME®: 10 nM thrombin (blue), 1 nM thrombin (yellow), 10% human serum (red), and blank (black). Little-to-no active thrombin was found in the human serum used in this study.

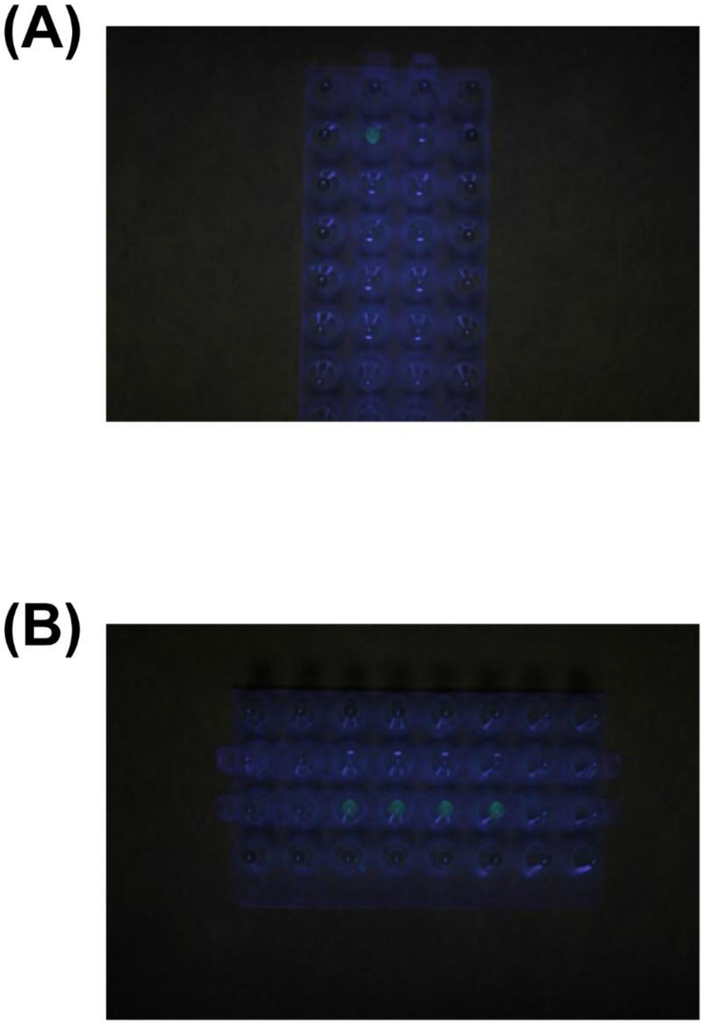

Figure S10. Uncropped images (A) of Figure 4A and (B) of Figure 4B, respectively.

(A)

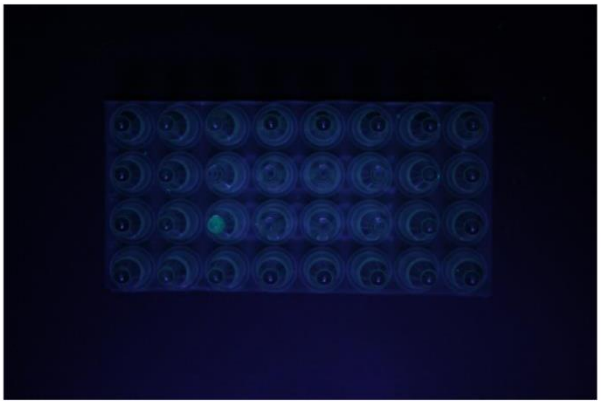

(B)

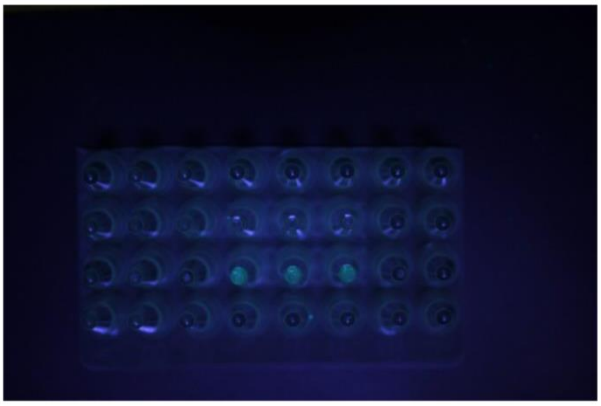

Figure S11. Uncropped images (A) of Figure 5A and (B) of Figure 5B, respectively.

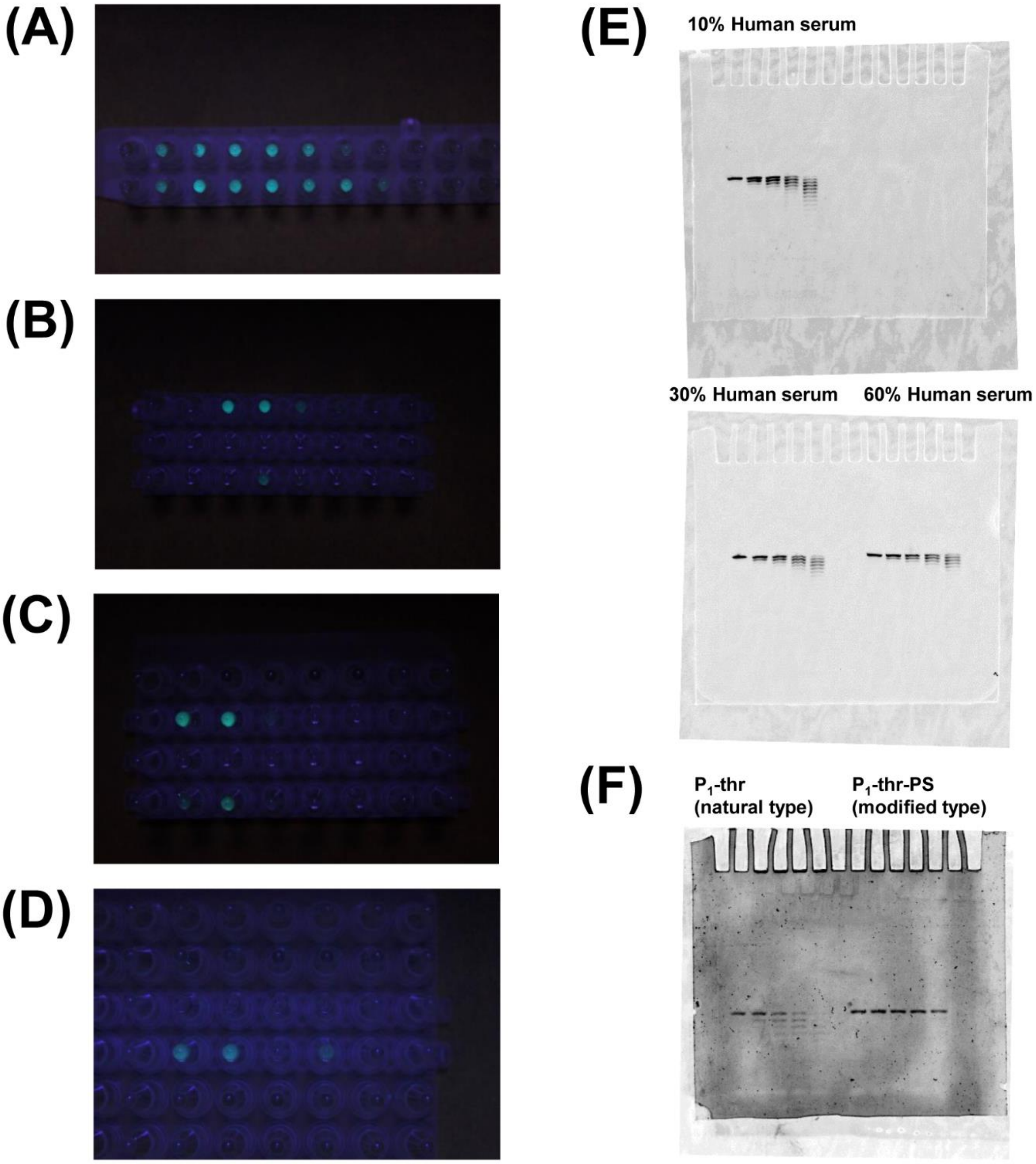

Figure S12. Uncropped images (A) of Figure S2, (B) of Figure S3A, (C) of Figure S3B, (D) of Figure S7, (E) of Figure S6A, and (F) of Figure S8A, respectively.
